# Validation of the 10-item Centre for Epidemiological Studies Depression Scale (CES-D-10) in Zulu, Xhosa and Afrikaans populations in South Africa

**DOI:** 10.1186/s12888-016-1178-x

**Published:** 2017-01-09

**Authors:** Emily Claire Baron, Thandi Davies, Crick Lund

**Affiliations:** 1 Alan J Flisher Centre for Public Mental Health, Department of Psychiatry and Mental Health, University of Cape Town, Office 32, Building B, 46 Sawkins Road, 7700 Rondebosch, Cape Town, South Africa; 2 Centre for Global Mental Health, Institute of Psychiatry, Psychology and Neuroscience, King’s College London, London, UK

**Keywords:** Validation, Depression, CES-D-10, South Africa, Prevalence, PHQ-9, Screening

## Abstract

**Background:**

The 10-item Centre for Epidemiological Studies Depression Scale (CES-D-10) is a depression screening tool that has been used in the South African National Income Dynamics Study (NIDS), a national household panel study. This screening tool has not yet been validated in South Africa. This study aimed to establish the reliability and validity of the CES-D-10 in Zulu, Xhosa and Afrikaans. The CES-D-10’s psychometric properties were also compared to the Patient Health Questionnaire (PHQ-9), a depression screening tool already validated in South Africa.

**Methods:**

Stratified random samples of Xhosa, Afrikaans and Zulu-speaking participants aged 15 years or older (*N* = 944) were recruited from Cape Town Metro and Ethekwini districts. Face-to-face interviews included socio-demographic questions, the CES-D-10, Patient Health Questionnaire (PHQ-9), and WHO Disability Assessment Schedule 2.0 (WHODAS). Major depression was determined using the Mini International Neuropsychiatric Interview. All instruments were translated and back-translated to English. Construct validity was examined using exploratory factor analysis with varimax rotation. Receiver Operating Characteristics (ROC) curves were used to investigate the CES-D-10 and PHQ-9’s criterion validity, and compared using the DeLong method.

**Results:**

Overall, 6.6, 18.0 and 6.9% of the Zulu, Afrikaans and Xhosa samples were diagnosed with depression, respectively. The CES-D-10 had acceptable internal consistency across samples (α = 0.69–0.89), and adequate concurrent validity, when compared to the PHQ-9 and WHODAS. The CES-D-10 area under the Receiver Operator Characteristic curve was good to excellent: 0.81 (95% CI 0.71–0.90) for Zulu, 0.93 (95% CI 0.90–0.96) for Afrikaans, and 0.94 (95% CI 0.89–0.99) for Xhosa. A cut-off of 12, 11 and 13 for Zulu, Afrikaans and Xhosa, respectively, generated the most balanced sensitivity, specificity and positive predictive value (Zulu: 71.4, 72.6% and 16.1%; Afrikaans: 84.6%, 84.0%, 53.7%; Xhosa: 81.0%, 95.0%, 54.8%). These were slightly higher than those generated for the PHQ-9. The CES-D-10 and PHQ-9 otherwise performed similarly across samples.

**Conclusions:**

The CES-D-10 is a valid, reliable screening tool for depression in Zulu, Xhosa and coloured Afrikaans populations.

## Background

Major depression is one of the leading causes of disease burden worldwide [1] and has clear economic implications [2, 3]. The South African Stress and Health (SASH) study, conducted between 2002 and 2004, investigated the national prevalence of mental disorders, and reported that nearly 10% of the population suffered from major depressive disorder at least once in their lifetime [4]. Yet, only one in four individuals with depression or anxiety receive treatment in South Africa [5].

Where mental health services are available, the use of indicated screening tools has been advocated as a way to detect individuals at risk of depression [6]. The timely identification of individuals displaying depressive symptoms is important, as it allows such individuals to be referred for mental health treatment services to prevent depressive symptoms from worsening into full clinical depression. The Centre for Epidemiological Studies Depression Scale (CES-D) is a 20-item screening tool, initially developed to detect depression in general populations [7]. It has been validated in a variety of settings, such as Zambia [8] and South Africa [9]. Subsequently, several shorter versions have been developed, including Andresen’s 10-item version (CES-D-10), generated through item-total correlations with the original 20-item CES-D [10]. Originally validated in the older population [11, 12], the CES-D-10 has good psychometric properties in both healthy and psychiatric populations [13, 14], and in adolescents [15]. In Andresen’s original study, a cut-off score of 8 or 10 on the CES-D-10 was identified as optimal to identify individuals at risk of depression. A few studies have since focused on the diagnostic validity of the CES-D-10, yet  all were conducted in the United States or in China, and cut-offs varied considerably, from 8 to 16 [11, 13, 14, 16]. The reliability and validity of the CES-D-10 has, however, never been investigated in South Africa.

The CES-D-10 has been used in the National Income Dynamics Study (NIDS), a South African national household panel study of 7300 households [17]. The study’s first wave was conducted in 2008 and another three waves were conducted to date, one every two years. At each survey, a range of economic, health and wellbeing data were collected from all household members of 15 years of age or more. While a few studies from the first waves of NIDS used the CES-D-10 as a longitudinal measure of depression severity [18–20], most have used a cut-off of 10 to classify participants at high risk of depression, as suggested by Andresen [21–24].

The aim of this study was to determine the reliability and validity of the CES-D-10 in three major South African languages: Zulu, Xhosa and Afrikaans. The psychometric properties of the CES-D-10 were also compared to those of the Patient Health Questionnaire (PHQ-9) [25]; another common screening tool for depression, already validated in primary health care patients in North West Province and in Gauteng in South Africa [26, 27].

## Methods

### Design

This validation study investigated the internal consistency, concurrent, construct and criterion validity of the CES-D-10 among Zulu, Xhosa and Afrikaans-speaking populations. These languages are the most commonly spoken in South Africa, according to the 2011 census [28]: 22.7% of the South African population speaks Zulu, 16.0% speaks Xhosa and 13.5% speaks Afrikaans. The study consisted of face-to-face interviews which included (1) basic demographic and economic questions; (2) depression and functioning screening instruments; and (3) the Mini International Neuropsychiatric Interview (MINI) 6.0 (Major Depressive Episode) [29].

### Measures

#### Demographic and socio-economic information

Basic demographic and socio-economic information covered age, gender, population group, marital status, education, employment status, personal income and assets owned. Household economic measures included type of dwelling, number of household members, as well as access to electricity, water and sanitation.

#### Centre for Epidemiological Studies Depression Scale (CES-D-10)

The CES-D-10 is a 10-item Likert scale questionnaire assessing depressive symptoms in the past week [10]. It includes three items on depressed affect, five items on somatic symptoms, and two on positive affect. Options for each item range from *“rarely or none of the time”* (score of 0) to *“all of the time”* (score of 3). Scoring is reversed for items 5 and 8, which are positive affect statements. Total scores can range from 0 to 30. Higher scores suggest greater severity of symptoms.

#### Patient Health Questionnaire – 9 item (PHQ-9)

The PHQ-9 is a 9-item screening measure for depression, where participants are asked to rate how often they were bothered by specific symptoms over the last two weeks [25]. Each item is scored from 0 (“Not at all”) to 3 (“Nearly every day”). Higher scores indicate greater symptoms of depression. The PHQ-9 has been validated in a range of settings and populations in low and middle-income countries [30], including South Africa [26].

#### Mini International Neuropsychiatric Interview (MINI) 6.0, Major Depressive Episode module

The presence of major depression was determined using the MINI 6.0 [29], which uses the DSM-IV criteria for major depressive episodes. It has been used as a gold standard in many cross-cultural studies, including in HIV-positive patients in South Africa [31, 32].

#### WHO Disability Assessment Schedule (WHODAS 2.0) (12-item)

Functional impairment was assessed using the WHODAS 2.0 [33]. It comprises 12 items with response options ranging from ‘No difficulty’ to ‘Extreme difficulty or unable to do’. The item-response-theory (IRT) based scoring was used, as set out in the WHODAS 2.0 Manual [34]: scores are percentages, with higher percentages suggesting greater impairment. The WHODAS 2.0 has undergone extensive validation, and has good reliability and validity across cultures and population groups [34].

All sections of the questionnaire, including the MINI assessment, were translated into Xhosa, Afrikaans and Zulu, and back translated to English, by six independent translators. The research team worked with the translators to assess the accuracy of each item, and to resolve discrepancies where these arose.

### Sample size

Three samples were recruited, one for each language. Given that the prevalence of individuals screening ≥10 or ≥15 on the CES-D-10 in the first wave of NIDS was 28 and 8% respectively in the Western Cape, and 32 and 5% in KwaZulu Natal,^1^ it was determined that a total of 300 participants per sample would be sufficient to analyse higher CES-D-10 scores, and have enough power to assess criterion validity. The sample size of validation studies included in a recent meta-analysis of the PHQ-9 [30] usually ranged from 150 to 600. The proposed sample therefore falls within the range of validation studies considered methodologically strong.

### Household sampling

Participants were recruited from two districts in South Africa: the City of Cape Town metro district and Ethekwini district in KwaZulu Natal, which encompasses both rural and urban areas. The ‘small area level’ (SAL) was used as the primary sampling unit from which to select households in the two districts. The SAL is the lowest level of geographic unit for which Census data is publically available, and is a manageable size in terms of population and land area. Population sizes vary across SALs, but usually range between 400 and 1000 individuals.

Only SALs classified as residential were included in the sampling base. SALs were selected for inclusion using systematic sampling, based on data from StatsSA. SALs were stratified by the most common home language, main population group (White, Black, Coloured, Indian), type of area (rural/urban) and most common income bracket. In South Africa, the term ‘coloured’ is not considered critical, and is used to describe an ethnic group composed primarily of persons of mixed race.

A total of six participants were recruited per SAL, with a maximum of two participants per household. The first household in each SAL was selected using a random starting point (created using a sampling algorithm on the Geographic Information System). Every third household was then selected from this starting point. Non-dwelling structures, such as shops, churches and museums, were skipped. Households were still included in the three count method when members were not at home or refused to participate.

This process was repeated until six participants per SAL were reached. If this could not be reached in a particular SAL, then the nearest predetermined oversampled SAL with the same settlement pattern was attempted, in order to reach the full complement of six participants. A total of 75 SALs were selected per sample, including an additional 50% of oversampled SALs.

### Participants

To be eligible, participants had to be aged 15 years or more, and be able to provide consent. Their home language had to be Xhosa, Afrikaans or Zulu, depending on the district, and be considered household members. This was defined as relatives or non-relatives who lived under the same roof or within the same compound, shared resources, and slept in the house for at least four nights a week. Live-in domestic workers and lodgers were regarded as separate households.

### Training

All fieldworkers conducting the interviews received one week of training by a registered counsellor (TD), on mental illness, administration of the tools, and methodological procedures. The first part of the training consisted of general psychoeducation on symptoms of depression and available treatments, and open discussions on the fieldworkers’ knowledge or experience of depression. The second part of the training included a back-translation of the translated MINI, as a cognitive testing exercise, to ensure the fieldworkers had a full understanding of the concepts of symptoms assessed in the MINI, and to ensure these corroborated with the translators’ translation. Fieldworkers were then trained to administer the MINI and the other screening tools, facilitated by role plays during which inter-rater reliability was also informally assessed. The depth of training on depression, in addition to the tool itself, was essential to ensure the accuracy of the fieldworkers’ diagnostic assessments, and ensure that the data collected were robust and the interpretation of results reliable. Finally, TD spent three days with each fieldwork team at the start of data collection, shadowing all interviews conducted to monitor the quality of the MINI assessment and of the accuracy of diagnoses made.

### Procedure

Xhosa and Afrikaans speaking participants were recruited from the City of Cape Town metro district and Zulu speaking participants from the Ethekwini district. Each sample of 300 participants was recruited by one team, comprising of two experienced, trained fieldworkers. Aerial maps of the SALs were printed and provided to the fieldworkers to navigate the SALs. The starting point for the SAL and non-dwelling structures were indicated on the maps. Fieldworkers first approached the households to determine that the language criterion was met. If a household member was present, eligible and agreed, he or she was asked to provide a list of all eligible members in the household, even if they were not present at the time of the visit. Two participants were then randomly selected, using the Dice method: a number was assigned to each eligible household member and an 18-faced dice was thrown to select the assigned number for individuals to be recruited. Appointments were made if selected individuals in the household were not present. A missed appointment was considered as a refusal.

Data were collected electronically, with the use of mobile devices. The interview was administered by the same fieldworkers involved in the recruitment process. The CES-D-10, PHQ9 and WHODAS 2.0 were administered separately from the socio-economic section and MINI 6.0 depression module, and by a different fieldworker, to avoid response bias. Each section of the interview was conducted in a private area of the participant’s home, away from other household members and the second fieldworker. Minors completed the interview in the presence of the consenting caregiver. The full interview lasted approximately 45 min.

### Statistical analysis

The data collected were transferred to Stata version 13, where analyses were conducted separately for each sample. Descriptive statistics were used to describe the socio-demographic characteristics of the participants, their screening scores and depression diagnosis. A review of kurtosis and skewness suggested that none of the scores on the CES-D-10, PHQ-9 or WHODAS 2.0 were normally distributed, so non-parametric tests and medians (interquartile range; IQR) were reported throughout the analysis. Probability weights were calculated to estimate the population-level prevalence of depression, taking into account the selection of eligible SALs among the districts, and the probability of a household being selected within an SAL and of an individual being selected within a household. Non-parametric independent tests were used to compare CES-D-10 scores between depressed and non-depressed participants. The internal reliability of the CES-D-10 and PHQ-9 were assessed using Cronbach’s Alpha. The CES-D-10’s convergent validity was determined by assessing its correlation with the WHODAS 2.0 and the PHQ-9. An exploratory factor analysis with varimax rotation was applied to investigate the construct validity of the CES-D-10, using the Kaiser Test and scree plot to identify latent dimensions of the scale. Finally, Receiver Operating Characteristics (ROC) curves were used to examine the CES-D-10 and PHQ-9’s criterion validity against the MINI 6.0. Optimal cut-off scores were identified as the best balance between sensitivity and specificity values, giving equal weight to both measures. The area under the ROC curve for the CES-D-10 was compared to that of the PHQ-9 using the DeLong method [35].

### Ethical considerations

This study was approved by the University of Cape Town’s Health Sciences Faculty Human Research Ethics committee (REF: 209/2016). Consent and assent forms were translated in all three languages and completed by all participants who agreed to participate. A R20 supermarket voucher was given to each participant, at the end of the interview. Participants who were diagnosed with depression were given a brochure on depression, and a list of local non-governmental organisations and toll-free numbers they could contact for counselling. Participants who reported suicidal behaviour were referred to the mental health nurse at a primary health care clinic of their choice. Suicide behaviours were considered present if participants answered ‘yes’ to the MINI 6.0 item (“Did you repeatedly consider hurting yourself, feel suicidal or wish that you were dead? Did you attempt suicide or plan a suicide?”), or answered ‘several days’ or more to PHQ9 item (“Thoughts that that you would be better off dead or of hurting yourself in some way”).

## Results

**Fig. 1:**
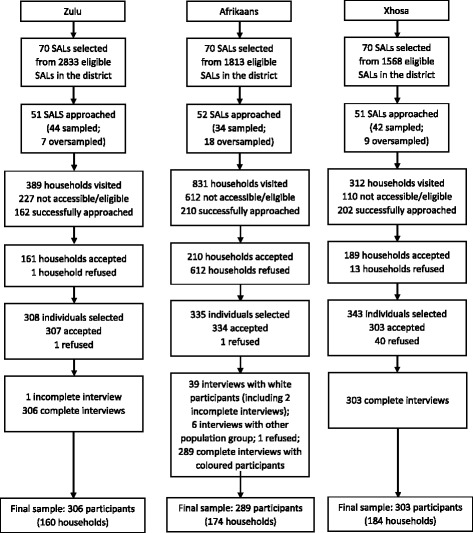
Recruitment process for the Zulu, Afrikaans and Xhosa samples

A total of 944 participants were recruited: 307 in the Zulu sample, 334 in the Afrikaans sample and 303 in the Xhosa sample. One participant in the Zulu sample and two in the Afrikaans sample did not complete the questionnaire (Fig. 1). The original intention was to recruit two types of population group in the Afrikaans sample: one third ‘white’ and two-thirds ‘coloured’, to be representative of the Afrikaans population in the district. However, the rate of refusals was high among the white population, and only 39 white participants were recruited. Given that the socio-economic characteristics of the white and coloured populations differed significantly in the study, the 39 white participants and 6 participants reporting to be other than coloured, were excluded from the analysis. To increase the Afrikaans coloured sample, additional participants were recruited from the remaining oversampled Afrikaans SALs. The final sample included in the analysis was therefore 306 for the Zulu sample, 289 in the coloured Afrikaans sample and 303 in the Xhosa sample.

### Sample demographic and socio-economic characteristics

The majority of participants across samples were recruited from urban (formal and informal) settlements (Table 1). Half of the Zulu sample (53.8%), and a third of the Afrikaans sample lived in formal houses. The majority of participants in the Afrikaans sample, however, lived in government housing. A third of the Xhosa sample also reported living in government houses, and nearly half in informal dwellings. The majority of households across samples reported having access to electricity, piped water and private flush toilet facilities inside or outside the dwelling.

**Table 1:** Socio-demographic and socio-economic characteristics of the three samples

	Zulu		Afrikaans		Xhosa	
	*N*	%	*N*	%	*N*	%
Age	306		288		303	
Median	33		47		32	
25^th^–75^th^ percentile	23–47		28–59		23–44	
Gender	306		289		303	
Male	95	31.1	85	29.4	98	32.3
Female	211	68.9	204	70.6	205	67.7
Population group	306		289		303	
African	306	100	0	-	303	100.0
Coloured	0	-	289	100.0		
Marital status	305		288		303	
Single	233	76.4	106	36.8	174	57.4
Living with partner but not married	16	5.3	2	0.7	46	15.2
Married and living together	21	6.9	123	42.7	56	18.5
Married but not living together	7	2.3	8	2.8	14	4.6
Divorced or widowed	28	9.1	49	17.0	13	4.3
Educational level	305		286		303	
No schooling/Grade 0–2	8	2.6	6	2.1	6	2.0
Primary school	51	16.8	67	23.4	31	10.2
Some secondary school	117	38.5	156	54.6	184	60.7
End of secondary school	94	30.9	39	13.6	68	22.4
Post-secondary school	33	10.9	17	5.9	9	3.0
Primary school	1	0.3	1	0.3	5	1.7
Employment	305		289		303	
Working for pay	62	20.3	50	17.3	105	34.7
Self-employed	4	1.3	6	2.1	9	3.0
Scholar/student	48	15.7	19	6.6	59	19.5
Homemaker	1	0.3	72	24.9	4	1.3
Long term sick or disabled	1	0.3	11	3.8	4	1.3
Retired	38	12.5	63	21.8	11	3.6
Unemployed and looking for a job	119	39.0	64	22.2	104	34.3
Unemployed but not looking for a job	32	10.5	4	1.4	7	2.3
Personal monthly income	306		289		303	
R0	107	35.0	105	36.3	98	32.4
R1-R500	51	16.7	15	5.2	47	15.5
R501-R1,000	30	9.8	23	8.0	27	8.9
R1001-R2000	61	19.9	98	33.9	57	18.8
R2000-R5000	18	5.9	20	6.9	64	21.1
R5001-R10000	6	2.0	12	4.2	7	2.3
R10001-R20000	6	2.0	5	1.7	2	0.7
More than R20,000	0	-	5	1.7	0	-
Don’t know/Refused	27	8.8	6	2.1	1	0.3
Personally own:	306		289		303	
A motor vehicle	13	4.3	31	10.7	12	4.0
A motorcycle	0	0	3	1.0	0	-
A computer/laptop	18	5.9	24	8.3	16	5.3
A cell phone	288	94.1	206	71.3	269	88.8
Settlement type	160		172		184	
Urban-formal	84	52.5	166	96.5	123	66.9
Urban-informal	49	30.6	6	3.5	61	33.1
Rural-traditional	27	16.9	0	-	0	-
Dwelling type	160		174		184	
Informal/backyard dwelling	24	15.0	4	2.3	81	44.0
RDP (government) house^a^	45	28.1	105	60.3	69	37.5
Flat	3	1.9	7	4.0	5	2.7
Formal house	86	53.8	58	33.3	25	13.6
Other	2	1.2	0	-	4	2.2
Electricity	160		174		184	
Yes	152	95.0	172	98.9	180	97.3
Source of drinking-water	158		174		184	
Piped water on site or in yard	129	81.7	170	97.7	118	64.1
Public tap	18	11.4	4	2.3	64	34.8
Borehole off site/communal	5	3.2	0	-	0	-
From neighbours	2	1.3	0	-	2	1.1
Other	4	2.5	0	-	0	-
Type of toilet	160		174		183	
Flush toilet inside dwelling (private)	91	56.9	164	94.3	78	42.6
Flush toilet outside dwelling (private)	3	1.9	7	4.0	56	30.6
Private bucket system or pit latrine	43	26.9	0	-	4	2.2
Communal flush toilet	5	3.1	2	1.1	21	11.5
Communal bucket or pit latrine	16	10.0	1	0.6	22	12.0
Other	2	1.2	0	-	2	1.1
Number of members aged 15+ years	160		174		184	
Median	4		4		2	
25^th^ – 75^th^ percentiles	2.5–5		2–5		2–4	

^a^The Reconstruction and Development Programme is a South African policy framework put in place by the African National Congress (ANC) government in 1994. Part of the RDP plan was to build 1,000,000 low-cost houses to overcome inadequate housing in urban townships and rural settlements

The majority of participants sampled were women (67.7–70.6% across samples). Participants in the Afrikaans sample were older (Mean = 44.7, SD = 17.72, range: 15–84) compared to participants in the Zulu sample (Mean = 33, SD = 15.63, range: 15–86) and Xhosa sample (Mean = 34.0, SD = 13.49, range: 15–77). A larger proportion of adolescents were recruited in the Xhosa sample (*N* = 27; 8.9%) compared to the Zulu and Afrikaans samples (2.3 and 2.4%, respectively). Most of the participants reported being single in the Zulu (76.4%) and Xhosa (57.4%) samples, while this group was a minority in the Afrikaans sample (36.7%). Nearly a third of the Zulu sample reported having reached the end of high school, which was the case only for 13.6% of the participants in the Afrikaans sample and 22.4% in the Xhosa sample. A minority reported having tertiary education across samples (4.7–11.1%). A fifth of the Zulu sample and over a third of the Xhosa sample reported being employed. Another 40% in the Zulu sample and 34% in the Xhosa reported being unemployed and looking for work. The remaining participants were mainly school or university students. On the other hand, participants in the Afrikaans sample consisted mostly of ‘stay at home’ individuals (looking after children or home; 24.9%), unemployed (23.6%) and retired (21.8%) individuals. A third of each sample reported not receiving a personal income. Remaining participants usually indicated earning less than R5,000 (US$ 320) per month.

### Prevalence of major depression

The prevalence of depression in the three samples and across demographic groups are reported in Table 2. A similar proportion of participants in the Zulu and Xhosa samples were diagnosed with depression (6.9%), but a much higher prevalence was found in the Afrikaans sample (18.0%). Only one adolescent, in the Afrikaans sample, was diagnosed with depression.

**Table 2:** MINI diagnosis of depression across demographic groups

	Zulu sample			Afrikaans sample			Xhosa sample		
	MINI diagnosis (*n* = 306)		*X* ^2^	MINI diagnosis (*n* = 289)		*X* ^2^	MINI diagnosis (*n* = 303)		*X* ^2^
	No (*n* = 285)	Yes (*n* = 21)		No (*n* = 237)	Yes (*n* = 52)		No (*n* = 282)	Yes (*n* = 21)	
Gender									
Male	93 (33%)	2 (10%)	4.9^a^	72 (30%)	13 (25%)	0.6	94 (33%)	4 (19%)	1.8
Female	192 (67%)	19 (90%)		165 (70%)	39 (75%)		188 (67%)	17 (81%)	
Age									
15–20	28 (10%)	1 (5%)	9.9^a^	25 (11%)	4 (8%)	12.0^a^	54 (19%)	1 (5%)	6.7
21–40	161 (56%)	9 (43%)		76 (32%)	18 (35%)		147 (52%)	10 (48%)	
41–60	73 (26%)	5 (24%)		77 (33%)	27 (52%)		71 (25%)	10 (48%)	
61 or older	23 (8%)	6 (28%)		58 (25%)	3 (6%)		10 (4%)	0	
Marital status									
Single	219 (77%)	14 (67%)	11.4^a^	87 (37%)	19 (37%)	13.0^a^	160 (57%)	14 (67%)	1.0
Living with partner	16 (6%)	0		0	2 (4%)		44 (16%)	2 (9%)	
Married (living together)	20 (7%)	1 (5%)		106 (45%)	17 (33%)		53 (19%)	3 (14%)	
Married (not living together)	7 (2%)	0		5 (2%)	3 (6%)		13 (5%)	1 (5%)	
Divorced or widowed	22 (8%)	6 (29%)		39 (16%)	10 (20%)		12 (4%)	1 (5%)	
Education level									
No schooling	8 (3%)	0	7.5	4 (2%)	2 (4%)	3.7	6 (2%)	0	2.6
Primary school	44 (15%)	7 (33%)		57 (24%)	10 (19%)		28 (10%)	3 (14%)	
Some secondary school	108 (38%)	9 (43%)		123 (53%)	33 (63%)		174 (62%)	10 (48%)	
End of secondary school	91 (32%)	3 (14%)		34 (15%)	5 (10%)		61 (22%)	7 (33%)	
Post-secondary school	1 (0%)	1 (5%)		16 (7%)	2 (4%)		13 (5%)	1 (5%)	
Employment status									
Working for pay	59 (21%)	3 (14%)	18.1^a^	45 (19%)	5 (10%)	9.8	100 (35%)	5 (24%)	5.2
Self-employed	2 (1%)	2 (9%)		5 (2%)	1 (2%)		9 (3%)	0	
Scholar/student	46 (16%)	2 (9%)		16 (7%)	3 (6%)		56 (20%)	3 (14%)	
Homemaker	1 (0%)	0		54 (23%)	18 (35%)		4 (1%)	0	
Long term sick/disabled	1 (0%)	0		8 (3%)	3 (6%)		4 (1%)	0	
Retired	32 (11%)	6 (29%)		57 (24%)	6 (12%)		10 (3%)	1 (5%)	
Unemployed (looking)	113 (40%)	6 (29%)		49 (21%)	15 (29%)		93 (33%)	11 (52%)	
Unemployed (not looking)	30 (11%)	2 (9%)		3 (1%)	1 (2%)		6 (2%)	1 (5%)	
Personal income									
R0	103 (36%)	4 (19%)	4.6	85 (37%)	20 (39%)	5.1	93 (33%)	5 (24%)	9.9
R1-R500	48 (17%)	3 (14%)		10 (4%)	5 (10%)		40 (14%)	7 (33%)	
R501-R1,000	27 (9%)	3 (14%)		17 (7%)	6 (12%)		27 (10%)	0	
R1001-R2000	54 (19%)	7 (33%)		93 (36%)	15 (19%)		51 (18%)	6 (29%)	
R2000-R5000	16 (6%)	2 (9%)		18 (8%)	2 (4%)		62 (22%)	2 (10%)	
More than R5000	11 (4%)	1 (5%)		19 (8%)	3 (6%)		8 (3%)	1 (5%)	
Dwelling type	(*n* = 142)	(*n* = 16)		(*n* = 136)	(*n* = 38)		(*n* = 169)	(*n* = 11)	0.5
Informal/backyard dwelling	21 (15%)	3 (19%)	5.1	1 (1%)	3 (8%)	9.0^a^	76 (45%)	5 (45%)	
RDP house	37 (26%)	8 (50%)		84 (62%)	21 (55%)		65 (38%)	4 (36%)	
Flat	3 (2%)	0		4 (3%)	3 (8%)		5 (3%)	0	
Formal house	81 (56%)	5 (31%)		47 (35%)	11 (29%)		23 (14%)	2 (18%)	

^a^significant at <0.05 level

None of the socio-demographic measures were associated with a diagnosis of depression in the Xhosa sample. In the Zulu sample, gender, age, marital status and employment status were associated with depression. A significantly higher proportion of participants with depression were women (90%), were aged 60 years or more (28%), were retired (39%), and were either divorced or widowed, compared to non-depressed participants (67, 8, 11 and 8%, respectively). A greater proportion of non-depressed participants reported being employed (21%) or studying (16%) compared to non-depressed participants (14 and 9% respectively).

In the Afrikaans sample, age and marital status were associated with depression, as well as dwelling type: a greater proportion of depressed participants were between 40 and 60 years old (52%), and living in informal settlements. More non-depressed participants reported being married and living together (45%) in comparison to depressed participants (33%).

Taking into account the sampling strategy, the weighted population prevalence of depression was 5.9% (95% CI 3.0–11.4) in the Zulu sample, 18.9% (95% CI 13.5%–25.7%) in the Afrikaans sample, and 6.9% (95% CI 4.0%–11.5%) in the Xhosa sample.

### Depression screening and functioning scales

Scores on the CES-D-10 were significantly higher for participants diagnosed with depression (Zulu: median = 15, IQR = 8; Afrikaans: median = 20, IQR = 11; Xhosa: median = 18; IQR = 6) compared to those who were not depressed (Zulu: median = 9, IQR = 6; Afrikaans: median = 3; IQR = 8; Xhosa: median = 4; IQR = 5). Adolescents did not have significantly different CES-D-10 scores compared to adults in the Zulu or Afrikaans samples, but had significantly lower CES-D-10 scores in the Xhosa sample (median = 3; IQR = 4) compared to adults (median = 4; IQR = 6) (*U* = 186937.0, *p* < .05).

The three scales had adequate inter-item reliability across samples (CES-D-10: 0.69–0.89; PHQ-9: 0.73–0.86; WHODAS 2.0: 0.74–0.84). CES-D-10 item-rest correlations were all above 0.37, 0.40 and 0.60 in the Zulu, Afrikaans and Xhosa samples, respectively, with the exception of items 5 (“I felt hopeful about the future”) and 8 (”I was happy”), which consistently had the lowest item-rest correlations, ranging from 0.03 to 0.30 and 0.04 to 0.52, respectively. Internal reliability and item-rest correlations for item 5 and 8 were higher in the Afrikaans sample.

### Concurrent validity of the CES-D-10

The correlation between the CES-D-10 and the other screening tools were all above .5 and statistically significant at the 0.001 level, besides the correlation between the CES-D-10 and the WHODAS 2.0, which was lower in the Xhosa sample (Rho = 0.37).

### Construct validity of the CES-D-10

The exploratory factor analysis suggests a two-factor solution in the Zulu and Xhosa samples, explaining 42.7 and 46.7% of the variance on the CES-D-10, respectively. A one-factor model in the Afrikaans sample was identified, explaining 51.6% of the variance. Item-factor correlations are shown in Table 3. All items pertaining to negative affect and somatic symptoms loaded highly on Factor 1 (0.55–0.71), and items 5 and 8, which refer to positive affect, either loaded highly on Factor 2 in the Zulu and Xhosa samples, or less well on Factor 1 in the Afrikaans sample.

**Table 3:** Varimax-rotated factor loadings (>0.3) and unique variances of CES-D-10 items

CES-D-10 items	Zulu		Afrikaans	Xhosa	
	Factor 1	Factor 2	Factor 1	Factor 1	Factor 2
1. I was bothered by things that usually don’t bother me	0.57		0.78	0.55	
2. I had trouble keeping my mind on what I was doing	0.57		0.83	0.66	
3. I felt depressed	0.64		0.83	0.76	
4. I felt that everything I did was an effort	0.68		0.71	0.61	
5. I felt hopeful about the future		0.69	0.36		0.80
6. I felt fearful	0.56		0.67	0.53	
7. My sleep was restless	0.55		0.70	0.64	0.30
8. I was happy		0.77	0.60		0.67
9. I felt lonely	0.71		0.80	0.69	
10. I could not “get going”	0.66		0.77	0.61	

### Criterion validity of the CES-D-10 and PHQ-9

**Fig. 2:**
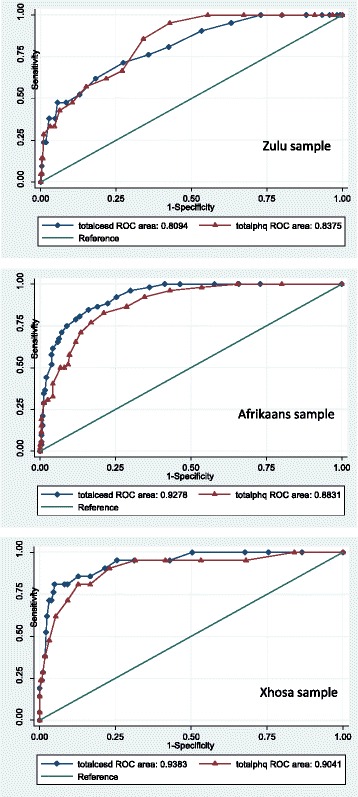
Receiving Operating Characteristics curves for the CES-D-10 and the PHQ-9

The CES-D-10 and PHQ-9 ROC curves are presented in Fig. 2. The area under the ROC curve (AUROC) for both screening tools were good to excellent across all three samples (0.81–0.94). In the Zulu sample, the AUROC for the CES-D-10 (0.81; 95% CI 0.71–0.90; <0.05) was slightly lower than that of the PHQ-9 (0.85; 95% CI 0.78–0.92; <0.05), but not significantly different. In the Afrikaans sample, the AUROC for the CES-D-10 was significantly higher (0.93; 95% CI 0.90–0.96; <0.05) than that of the PHQ-9 (0.88; 95% CI 0.84–0.93; <0.05) (*X* ^2^ = 0.43, *p* < 0.05). The AUROC for the CES-D-10 (0.94; 95% CI 0.89–0.99; <0.05) was also higher in the Xhosa sample, compared to the PHQ-9 (0.90; 95% CI 0.83–0.98; <0.05), but this difference was not statistically significantly.

#### CES-D-10

The sensitivity and specificity of each cut-off point on the CES-D-10 and PHQ-9 are presented in Table 4. In the Zulu sample, a cut-off of 12 on the CES-D-10 presented the most balanced sensitivity (71.4%) and specificity (72.6%), correctly classifying 72.6% of participants. However, the positive predictive value (PPV) was very low, suggesting that only 16.1% of those who scored 12 or above on the CES-D-10 were depressed. This is due to the low prevalence depression in this sample (6.6%). In the Xhosa sample, where the prevalence of depression was also low, a cut-off of 10 on the CES-D-10 presented the most balanced sensitivity (85.7%) and specificity (87.2%), correctly classifying 84.1% of participants. Again, the PPV was very low (33.3%). However, a higher cut-off of 13 still had adequate sensitivity (81.0%) and specificity (95.0%), though less balanced, and an acceptable PPV (54.8%), altogether correctly classifying 94.1% of the sample. Finally, in the Afrikaans sample, a cut-off of 11 on the CES-D-10 presented the most balanced sensitivity (84.6%) and specificity (84.0%), correctly classifying 84.1% of participants. The PPV was higher in this sample, with 53.7% of participants with a CES-D-10 score of 11 or above having a diagnosis of depression.

**Table 4:** Optimal cut-off scores on the CES-D-10 and PHQ-9 for the detection of major depressive disorder

Cut-off score	Zulu					Afrikaans					Xhosa				
	Sensitivity	Specificity	PPV	NPV	Correctly classified	Sensitivity	Specificity	PPV	NPV	Correctly classified	Sensitivity	Specificity	PPV	NPV	Correctly classified
CES-D-10															
≥ 5	100.0	11.9	7.7	100.0	18.0	100.0	58.7	34.7	100.0	66.1	95.2	57.1	14.2	99.4	59.7
≥ 6	100.0	20.0	8.4	100.0	25.5	98.1	63.7	37.2	99.3	69.9	95.2	68.8	18.5	99.5	70.6
≥ 7	100.0	27.0	9.2	100.0	32.0	96.2	70.0	41.3	98.8	74.7	95.2	74.5	21.7	99.5	75.9
≥ 8	95.2	36.8	10.0	99.1	40.9	92.3	74.7	44.4	97.8	77.9	90.5	78.4	23.8	99.1	79.2
≥ 9	90.5	46.7	11.1	98.5	49.7	88.5	77.7	46.5	96.8	79.6	85.7	83.3	27.7	98.7	83.5
≥ 10	81.0	57.5	12.3	97.6	59.2	85.5	81.0	50.0	96.5	82.0	85.7	87.2	33.3	98.8	87.1
**≥ 11**	76.2	64.2	13.6	97.3	65.0	**84.6**	**84.0**	**53.7**	**96.1**	**84.1**	81.0	90.8	39.5	98.5	90.1
**≥ 12**	**71.4**	**72.6**	**16.1**	**97.2**	**72.6**	80.8	86.9	57.5	95.4	85.8	81.0	91.8	42.5	98.5	91.1
**≥ 13**	61.9	81.8	20.0	96.7	80.4	78.9	88.2	59.4	95.0	86.5	**81.0**	**95.0**	**54.8**	**98.5**	**94.1**
≥ 14	52.4	87.0	22.9	96.1	84.6	76.5	91.1	65.0	94.3	88.2	76.2	95.4	55.2	98.2	94.1
PHQ-9															
≥ 4	100.0	32.6	9.9	100.0	37.3	96.2	57.7	33.6	98.5	64.7	95.2	58.5	14.6	99.4	61.1
≥ 5	100.0	44.3	11.7	100.0	48.2	92.3	65.8	37.5	97.5	70.6	95.2	68.4	18.4	99.5	70.3
≥ 6	95.2	57.1	14.1	99.4	59.7	86.5	71.4	40.2	96.0	74.1	90.5	77.0	22.6	99.1	77.9
**≥ 7**	85.7	66.0	15.7	98.4	67.3	**82.7**	**79.1**	**46.7**	**95.4**	**79.7**	81.0	83.3	26.6	98.3	83.2
**≥ 8**	**66.7**	**73.1**	**15.4**	**96.7**	**72.6**	77.0	82.9	50.0	94.2	81.8	**81.0**	**87.2**	**32.1**	**98.4**	**86.8**
≥ 9	61.9	78.0	17.3	96.5	76.9	71.2	86.3	53.6	93.1	83.6	71.4	90.8	36.6	97.7	89.4
≥ 10	57.1	84.8	21.8	96.4	82.8	65.4	88.0	54.8	92.0	83.9	61.9	94.7	46.4	97.1	92.4
≥ 11	47.6	89.4	25.0	95.9	86.5	57.7	90.2	56.6	90.6	84.3	47.6	96.8	52.6	96.1	93.4
≥ 12	42.9	93.6	33.3	95.7	90.1	51.9	90.6	55.1	89.5	83.6	38.1	98.2	61.5	95.5	94.1

*PPV* Positive predictive value, *NPV* Negative predictive value

Optimal cut-off scores are indicated in bold

#### PHQ-9

Sensitivity and specificity values on the PHQ-9 were generally lower than on the CES-D-10 in all three samples. In the Zulu and Xhosa sample, a cut-off of 8 on the PHQ-9 provided the best balance of sensitivity and specificity (Zulu: 66.7 and 73.1%; Xhosa: 81.0 and 87.2%). While 72.6 and 86.8% of participants were correctly classified in the Zulu and Xhosa samples, respectively, the PPV was also low (15.4 and 32.1%, respectively). Selecting another cut-off score on the PHQ-9 did not improve the PPV without being detrimental to sensitivity or specificity values. On the other hand, a cut-off of 7 on the PHQ-9 in the Afrikaans sample provided the best balance of sensitivity (82.7%) and specificity (79.1%), correctly classifying 79.7% of participants; 46.7% of participants screening positive using this cut-off were actually depressed.

## Discussion

The present study sought to investigate the reliability and validity of the CES-D-10 among three language groups in South Africa.

The sampling strategy allowed us to recruit a representative sample of the Xhosa, Zulu and coloured Afrikaans population in the Cape Town Metro and Ethekwini districts, so a population weighted prevalence of depression could be calculated. The estimated population prevalence of 5.9% in the Zulu and 6.9% in the Xhosa populations are similar to the national 12-month prevalence for major depression (4.9%) reported in the SASH study [36]. The estimated population prevalence of 18% in the coloured Afrikaans population was relatively high. Interestingly, the odds of suffering from a mood disorder in Williams et al [36]’s study were also higher in the Coloured community, compared to the White or African communities, but this finding was not statistically significant. Unfortunately, too few adolescents were recruited, and almost none reported having depression, so it was not possible to estimate the population prevalence of depression among adolescents. For ethical reasons, the adolescents’ caregivers were present during the interview. It is therefore possible that the very low prevalence among adolescents in this study may also have been due to a lack of confidentiality when providing their responses.

Scores on the CES-D-10 differed from those reported in the first wave of NIDS. In the present study, the proportion of participants screening ≥10 and ≥15 was consistently higher in the Zulu and Afrikaans samples. The proportion of participants in the Xhosa sample scoring ≥10 (17.8%) was lower than the 28% figure reported by NIDS in the Western Cape; however the proportion scoring ≥15 was very similar (approximately 8%). The differences in CES-D-10 scores reported here may be due to the relatively small and perhaps less representative sample in this study, in comparison with the NIDS sample.

ROC curves suggested that the CES-D-10 is an adequate screening tool to identify individuals at risk of depression. AUROC values were all above the minimum value of .75, which is considered clinically significant [37]. In the Zulu sample, a cut-off of 12 on the CES-D-10 seem to be the most appropriate to indicate high risk of depression, whereas a cut-off of 11 in the Afrikaans sample and 13 in the Xhosa sample were most suitable. Alternatively, a cut-off of 12 may be appropriate in the South African context, as it provides relatively acceptable sensitivity and specificity across all three language groups.

The present study suggests that a cut-off of 10 to indicate high risk of depression, as suggested by Andresen et al. [10], may not be optimal, especially if the screening tool is to be used in clinical settings, which are already overburdened in South Africa. Indeed, if a cut-off of 10 were used, nearly half of the Zulu sample and one third of the Afrikaans sample would be considered at high risk for depression. Also, misclassification of individuals into high or low risk for depression in the Zulu sample would increase from 27.4% (at a cut-off of 12) to 40.8%, and from 5.9% (at a cut-off of 13) to 12.9% in the Xhosa sample. The difference in misclassification would be less striking in the Afrikaans sample, but would still increase from 15.9% (at a cut off of 11) to 18.0%.

The CES-D-10 performed well in relation to the PHQ-9 and the WHODAS 2.0, suggesting adequate concurrent validity. The internal consistency of the CES-D-10 was also acceptable in the Afrikaans and Xhosa samples, though slightly lower in the Zulu sample. The internal reliability and exploratory factor analysis both suggest that the two positive affect items do not fit well with the other items in the tool, and constitute a second dimension. This supports previous evidence on the internal structure of the CES-D-10 among adolescents [15] and the older population in Asia [12, 13], suggesting that the CES-D-10 consists of a depressed affect dimension (including somatic symptoms) and positive affect dimension. In addition, item 5 (hopefulness) consistently performed poorly in comparison to item 8 in the present study. This was also reported in Bradley et al [15]’s validation study among adolescents, where they cautioned about conceptualising hopefulness as a positive affect concept. The order of the questions may also have explained the difference in the performance of the two items, as participants may have been confused by the first positive statement, after having answered a series of negative statements.

In comparison to the original 20-item version of the CES-D, the CES-D-10 has clear benefits. The present study suggests that the shorter version of the tool is still reliable and valid in assessing clinically significant depressive symptoms among the general Xhosa, Zulu and Afrikaans populations in South Africa. As a shorter instrument, the CES-D-10 is less time consuming to administer and therefore more feasible to use in both research and clinical settings, such as part of larger screening activities integrated in health services to identify and refer at-risk individuals.

Overall, the PHQ-9 also performed well across the three samples. A cut-off of 7 or 8 on the PHQ-9 in the present study was lower than the cut-off of 9 suggested among chronic care patients in the Northwest Province of south Africa [26] and a cut-off of 10 identified among patients attending a high HIV-burdened primary care clinic in Johannesburg [27]. Altogether, the psychometric properties of the CES-D-10 and PHQ-9 were similar across all three samples. Though the AUROC was only slightly higher for the CES-D-10 in the Afrikaans sample, the cut-offs identified on the CES-D-10 consistently generated higher sensitivity, specificity and PPV compared to the PHQ-9 cut-offs. Results therefore suggest that the performance of the CES-D-10 as a screening tool is on par with, if not slightly stronger than the PHQ-9.

Given that both instruments comprise of 10 items and should take the same time to administer, the authors recommend using either screening tool to assess depressive symptoms in the future. Despite the CES-D-10 having slightly stronger psychometric properties than the PHQ-9 in the present study, the poorer performance of the two CES-D-10 positive affect items may lead researchers and clinicians to give preference to the PHQ-9, however.

### Limitations

Limitations of the study should be noted. First, it was not possible to assess the cultural differences in the CES-D-10’s performance among the Afrikaans-speaking population in the Western Cape, given the small number of non-coloured individuals recruited. The difficulty in recruiting from the white population was also noted in previous waves of NIDS, and is not specific to the present study. However, given the differences in socio-demographic and economic characteristics found across the different Afrikaans-speaking groups in this study, the exclusion of non-coloured participants from the analysis meant that the remaining Afrikaans sample was more culturally homogeneous, and stronger interpretations of the findings could be made for this specific population.

The different cut-offs identified across the three samples suggest that generalising the present findings to other linguistic groups within South Africa may not be possible. Great care was taken in the translation and back-translation of the tool, so it is unlikely that any differences found between the three samples were due to translation errors. Instead, these are likely to reflect differences in the perception and experience of depression, and in the idioms of distress used among different South African populations [38].

Second, the prevalence of depression measured by the MINI 6.0 was relatively low in the Zulu and Xhosa samples, which weakens the inferences that can be made based on the results. Nonetheless, the results corroborate previous evidence on the internal structure of the scale, suggesting that psychometric properties of the CES-D-10 in these samples are reasonably robust.

Finally, despite the sampling methodology, gender was not proportionally distributed, and samples consisted predominantly of women. Results suggest women reported higher CES-D-10 scores and were more likely to be depressed compared to men in the Afrikaans and Xhosa samples. This finding corroborates results from the SASH study suggesting that women had a higher lifetime prevalence of depression or other mood disorders in South Africa [4]. This likely inflated the overall sample and estimated population prevalence of depression reported in this study, and may have affected the performance of the CES-D-10 in relation to the MINI. However, the gender distribution were relatively consistent across the three samples, so this cannot explain the disproportion in the prevalence of major depression reported among the Zulu, Xhosa and Afrikaans samples.

## Conclusion

The findings suggest that the CES-D-10 has good psychometric properties in Zulu, coloured Afrikaans and Xhosa-speaking populations, similar to those of the PHQ-9. The CES-D-10 is therefore an adequate tool to identify individuals at risk for depression among these populations. Given that different cut-offs were found for the three populations, the validation of the CES-D-10 in the other South African language groups may be required to identify language-specific cut-offs. Test-retest reliability was beyond the scope of this study, but would be useful for future studies to investigate the reliability of the CES-D-10 in the three languages. Through greater certainty about the validity of the CES-D-10, this study has the potential to contribute to substantial new knowledge on a range of socioeconomic predictors and consequences of depression in the unique NIDS longitudinal dataset.

## Abbreviations

AUROC: Area under the Receiver Operating Characteristics curve
CES-D: Centre for Epidemiological Studies Depression Scale
CES-D-10: Centre for Epidemiological Studies Depression Scale – 10 item version
CI: Confidence interval
DSM-IV: Diagnostic and Statistical Manual of Mental Disorders – fourth edition
IQR: Interquartile range
MINI: Mini International Neuropsychiatric Interview
NIDS: National income dynamic study
NPV: Negative predictive value
PHQ-9: Patient Health Questionnaire – 9 item version
PPV: Positive predictive value
ROC: Receiver Operating Characteristics
SAL: Small area level
SASH: South African Stress and Health study
WHODAS: WHO Disability Assessment Schedule.

## Footnotes

1 Budlender D (2015). Prevalence of poor mental health: Evidence from NIDS (short report).
